# Human iPSC-Derived MSCs Induce Neurotrophic Effects and Improve Metabolic Activity in Acute Neuronal Injury Models

**DOI:** 10.1523/JNEUROSCI.0606-24.2024

**Published:** 2024-11-04

**Authors:** Keiji Kawatani, Genesis Omana Suarez, Ralph B. Perkerson, Ephraim E. Parent, Toshihiko Nambara, Joshua A. Knight, Tammee M. Parsons, Kshama Gupta, Francis Shue, Alla Alnobani, Prasanna Vibhute, Hancheng Cai, Hugo Guerrero-Cázares, John A. Copland, Alfredo Quiñones-Hinojosa, Takahisa Kanekiyo

**Affiliations:** ^1^Department of Neuroscience, Mayo Clinic, Jacksonville, Florida 32224; ^2^Neuroscience Graduate Program, Mayo Clinic Graduate School of Biomedical Sciences, Mayo Clinic, Jacksonville, Florida 32224; ^3^Center for Regenerative Biotherapeutics, Mayo Clinic, Jacksonville, Florida 32224; ^4^Departments of Radiology, Mayo Clinic, Jacksonville, Florida 32224; ^5^Cancer Biology, Mayo Clinic, Jacksonville, Florida 32224; ^6^Neurosurgery, Mayo Clinic, Jacksonville, Florida 32224

**Keywords:** iPSC, MSC, neuron, neurotrophic effect, stem cell therapy

## Abstract

Mesenchymal stromal cell (MSC) therapy has regenerative potentials to treat various pathological conditions including neurological diseases. MSCs isolated from various organs can differentiate into specific cell types to repair organ damages. However, their paracrine mechanisms are predicted to predominantly mediate their immunomodulatory, proangiogenic, and regenerative properties. While preclinical studies highlight the significant potential of MSC therapy in mitigating neurological damage from stroke and traumatic brain injury, the variability in clinical trial outcomes may stem from the inherent heterogeneity of somatic MSCs. Accumulating evidence has demonstrated that induced pluripotent stem cells (iPSCs) are an ideal alternative resource for the unlimited expansion and biomanufacturing of MSCs. Thus, we investigated how iPSC-derived MSCs (iMSCs) influence properties of iPSC-derived neurons. Our findings demonstrate that the secretome from iMSCs possesses neurotrophic effects, improving neuronal survival and promoting neuronal outgrowth and synaptic activity in vitro. Additionally, the iMSCs enhance metabolic activity via mitochondrial respiration in neurons, both in vitro and in mouse models. Glycolytic pathways also increased following the administration of iMSC secretome to iPSC-derived neurons. Consistently, in vivo experiments showed that intravenous administration of iMSCs compensated for the elevated energetic demand in male mice with irradiation-induced brain injury by restoring synaptic metabolic activity during acute brain damage. ^18^F-FDG PET imaging also detected an increase in brain glucose uptake following iMSC administration. Together, our results highlight the potential of iMSC-based therapy in treating neuronal damage in various neurological disorders, while paving the way for future research and potential clinical applications of iMSCs in regenerative medicine.

## Significance Statement

Regenerative biotherapeutics using MSCs have emerged as a promising intervention for treating various neurological diseases. Our study explored the potential beneficial effects of human iPSC-derived MSCs (iMSCs) on neurons. We demonstrated that molecules secreted into the culture medium by iMSCs enhance regenerative capabilities by improving neuronal survival, growth, and metabolic activity, as well as synaptic functions, in human iPSC-derived neurons. Mouse experiments also suggested the potential of iMSC therapy to mitigate synaptic mitochondrial dysfunction and enhance brain glucose uptake during acute radiation-induced brain injury, steps that contribute to restoring normal neuronal function. Our results highlight that iMSCs may be a promising alternative cell product for treating neuronal damage, overcoming the inconsistent efficacy of somatic MSCs due to cell variability.

## Introduction

Mesenchymal stromal cell (MSC) is a specific adherent cell population isolated from various organs such as bone marrow, adipose tissues, and umbilical cord. MSCs are characterized by expressions of CD105, CD73, and CD90, and the ability to differentiate into osteoblasts, adipocytes, or chondrocytes (Dominici et al., 2006; Pendleton et al., 2013). Emerging evidence suggests that MSC therapy offers regenerative potential, given its ability to differentiate into various cell types, including bone, muscle, and neurons, and its paracrine mechanisms that suppress immune responses, promote tissue regeneration, and stimulate cell growth (Horwitz, 2003; Tropel et al., 2006; Wei et al., 2013; Han et al., 2022). MSCs also exhibit neurotrophic activities through multiple mechanisms, including the secretion of neurotrophic/proangiogenic factors, enhanced neurogenesis and remyelination, immune modulation, and differentiation into neurons and oligodendrocytes (Badyra et al., 2020). Indeed, the application of MSC therapy in neurological disorders has extended beyond preclinical models to clinical trials, aiming to treat acute neuronal damages following stroke and traumatic brain injury, as well as chronic neurodegenerative diseases such as Alzheimer's disease and amyotrophic lateral sclerosis (Horwitz, 2003; Baak et al., 2022; Karvelas et al., 2022). We are also conducting clinical trials using adipose tissue-derived MSCs for epilepsy (NCT06280092) and glioblastoma (NCT05789394; Ramos-Fresnedo et al., 2023). MSC therapy represents a promising avenue for regeneration and neuroprotection. Despite its promise in preclinical studies, the efficacy of MSC therapy remains controversial in clinical trials. Although clinical studies have demonstrated the safety of MSC therapy, MSCs have not been commercialized as therapeutic products due to concerns about reproducibility (Levy et al., 2020). MSCs derived from different tissue sources and donors exhibit variability, presenting challenges such as the generation of antibodies against allogeneic donor MSCs and their immune rejection (Ankrum et al., 2014; Costa et al., 2021; Mabuchi et al., 2021). Thus, developing novel approaches for MSC therapy is desired to improve reproducibility and minimize heterogeneity. Although the use of MSCs from bone marrow, adipose tissue, or umbilical cord blood represents a valuable strategy, there are several limitations, including loss of plasticity during expansion and limited amplification capacity of harvested cells. Alternative cell sources are needed to establish a scalable and effective supply chain for MSC-based therapy. In this context, induced pluripotent stem cells (iPSCs) have emerged as an ideal cellular resource for unlimited expansion and biomanufacturing. iPSCs, a type of pluripotent stem cell, can be derived from nonpluripotent cells, including fibroblasts and blood cells, by introducing specific transcription factors. This allows us to model neurological disorders and explore the potential of cell therapy using human cells. As such, we have successfully developed a protocol for differentiating iPSCs into MSCs (Burgess et al., 2023).

Here, we investigated how iPSC-derived MSCs (iMSCs) and their secretome influence iPSC-derived neurons, demonstrating their beneficial effects on neuronal survival, morphology, synaptic activity, and metabolism. We also show that intravenous administration of iMSCs ameliorates impaired brain metabolism in a mouse model of irradiation-induced acute brain injury. Our study provides new evidence for the therapeutic potential of iMSCs and their derivatives in treating various neurological diseases.

## Materials and Methods

### Generation and maintenance of iPSCs

Human iPSCs were generated from two healthy individuals (#1: MC0192; female, 83  years old and #2: MC0039, male, 73 years old) and cultured in TeSR-E7 complete medium (STEMCELL Technologies) on Matrigel-coated (Corning) dish as reported previously (Zhao et al., 2017). The iPSCs were cultured in mTeSR1 complete medium (STEMCELL Technologies) and passaged with Dispase (STEMCELL Technologies) every 6–9 d and subjected to treatment with ROCK inhibitor Y27632 (Sigma-Aldrich) for the first day. Karyotyping of the iPSC clones was performed by Mayo Clinic Genomics Core Facility.

### Differentiation of iPSCs into MSCs

Embryoid bodies (EBs) were generated from iPSCs (MC0192) dispensed to single cells using Accutase solution (STEMCELL Technologies) by culturing in 96-well round-bottomed culture plates (1.5 × 10^3^ cells/well) in induction medium composed of IMDM (Invitrogen), 20% KnockOut serum replacement (Invitrogen), 0.1 mM nonessential amino acid (Invitrogen), and 0.1 mM 2-mercaptoethanol (Invitrogen), with 10 µM ROCK inhibitor for 2 d. EBs were transferred on 6-well plates (4–5 EBs/well), cultured for an additional 3 d in induction medium, and passaged on to 1% Gelatin-coated 6-well plate. After 3 d, the medium was changed to the differentiation medium composed of IMDM (Invitrogen), 20% KnockOut serum replacement (Invitrogen), 0.1 mM nonessential amino acid (Invitrogen), 0.1 mM 2-mercaptoethanol (Invitrogen), and 10 ng/ml TGFβ1 (R&D). After 3 d, the media was replaced with regular MSC maintenance media composed of α-MEM (Invitrogen), 5% FBS, and GlutaMAX (Invitrogen). After passaging three times, FACS using Human MSC Analysis Kit (BD Stemflow) confirmed that iMSCs expressed >95% positivity for markers CD73, CD90, CD105, and <5% positivity for markers CD34, CD45, CD11b, CD19, and HLA-DR. Trilineage differentiation potential of iMSCs were also confirmed using oil red O staining for adipogenesis, alizarin red S staining for osteogenesis, and Alcian blue staining for chondrogenesis using StemPro Differentiation Kits (Invitrogen; Burgess et al., 2023; Gupta et al., 2024). To prepare the conditioned culture media, iMSCs were cultured in BrainPhys Neuronal Medium (STEMCELL Technologies) for 1 d. The collected conditioned media was centrifuged at 300 × *g* for 5 min to remove cell debris, and supernatant media was preserved at −80°C before being used for the experiments.

### Differentiation of iPSCs into NPCs and neurons

Neural precursor cells were differentiated from iPSCs (MC0039) using the STEMdiff SMADi Neural Induction Kit (STEMCELL Technologies) as previously described (Kawatani et al., 2023). Briefly, dissociated iPSCs (3.0 × 10^6^ cells/well) were transferred onto 24-well AggreWell 800 plates (STEMCELL Technologies) and cultured in STEMdiff SMADi Neural Induction Medium supplemented with 10 μM Y27632. Half of the medium in each well was exchanged every 2 d. On Day 8, the embryoid bodies were transferred onto Matrigel-coated 6-well plates and cultured in the induction medium for another 5 d to induce neural rosette formation. On Day 15, the neural rosettes were isolated from the surrounding EB-derived flat cells. The small clumps of isolated neural rosettes were transferred onto Matrigel-coated plates and cultured in the induction medium for additional 5–7 d. The neural rosettes were dissociated into single cells with TrypLE Express (Thermo Fisher Scientific) and replated onto the Matrigel-coated 24-well plate and cultured in the induction medium supplemented with 10 μM Y27632. The culture medium was switched to STEMdiff SMADi Neural Progenitor Medium (STEMCELL Technologies) 1 d after the replating. NPCs were passed every 5–7 d with daily medium change, followed by cryopreservation with STEMdiff Neural Progenitor Freezing Medium (STEMCELL Technologies). NPCs with passage numbers between 3 and 7 were cultured on the Matrigel-coated 24-well plate in STEMdiff Forebrain Neuron Differentiation Medium (STEMCELL Technologies) for 5–7 d. Dissociated NPCs were cultured on poly-ʟ-ornithine (PLO, Sigma)/laminin (Sigma-Aldrich)–coated plates in BrainPhys Neuronal Medium (STEMCELL Technologies) supplemented with 10 μM Y27632 for the differentiation into neurons. Half of the medium in each well was changed twice a week.

### Irradiation and mitochondrial respiratory inhibition in iPSC-derived neuron

For in vitro radiation experiments, iPSC-derived NPCs were seeded at a density of 3.0 × 10⁴ cells/well onto glass coverslips and cultured in BrainPhys Neuronal Medium (STEMCELL Technologies), supplemented with 10 μM Y27632 to support cell survival. After 1 week of neuronal maturation, cells were irradiated with a 1 Gy dose using an XRad160 biological irradiator (Precision X-Ray) in the presence either control medium or iMSC CM. Five days postirradiation, neurons were fixed for downstream immunocytochemical analyses to assess neuronal morphology and survival. For mitochondrial respiratory inhibition experiments, iPSC-derived NPCs were seeded at a density of 1.0 × 10⁴ cells/well in 96-well glass-bottom plates (Cellvis) and cultured in BrainPhys Neuronal Medium with 10 μM Y27632. After 1 week of maturation, the iPSC-derived neurons were exposed to 0.1 µM antimycin A (Sigma), an inhibitor of mitochondrial complex III, in the presence of either control medium or iMSC CM for an additional 2 weeks. The medium was changed by replacing half of the volume twice a week to maintain optimal culture conditions.

### Immunocytochemistry

Immunocytochemistry was performed as previously described (Kawatani et al., 2023) with some modifications. Cells were fixed with 4% paraformaldehyde in PBS and permeabilized with 0.2% Triton X-100 (Sigma-Aldrich) in PBS for 15 min. After blocking with 1% bovine serum albumin (BSA, Roche) in PBS for 1 h, the cells were incubated overnight at 4°C with primary antibodies against βIII-tubulin (1:500, #ab78078, Abcam). The cells were washed with PBS and incubated for 2 h with Alexa Fluor 488- or Alexa Fluor 594-conjugated secondary antibodies (Thermo Fisher Scientific), followed by nuclei counterstaining with DAPI (300 nM, #D1306, Thermo Fisher Scientific). The images were taken with an LSM880 inverted confocal microscope (Carl Zeiss). For neuronal irradiation and antimycin A treatment, images were acquired by Keyence fluorescence microscopy (model BZ-X, Keyence). To capture high-resolution images of large axonal areas, the automatic stitching function was utilized. Neurons were counted using the Analyze Particle feature in ImageJ (Fiji).

### Coculture system of iPSC-derived neurons and iMSCs in trans-well chambers

The dissociated NPCs (1.0 × 10^3^ cells/well) were seeded onto 96-well plates (Cellvis) and cultured in BrainPhys Neuronal Medium for 1 week. iMSCs (2 × 10^4^/well) with passage between 3 and 5 were then plated on cell culture inserts with 0.4-µm-diameter pores (Corning). The neurons were cocultured with the insert containing iMSCs in DMEM/F12 (Thermo Fisher Scientific). After 7 d of the coculture, neurons were fixed and stained with neuronal marker βIII-tubulin and DAPI as described above. The images were taken with an LSM880 inverted confocal microscope (Carl Zeiss). βIII-tubulin positive cells were counted using ImageJ (Fiji).

### Neuronal morphology analysis

The NPCs (1.0 × 10^3^ cells/well) were seeded onto 96-well plates (Cellvis) and cultured in BrainPhys Neuronal Medium for 1 week. The neurons were then cultured in normal neuron media or conditioned media of iMSCs for an additional 3 weeks. The neurons were fixed and stained with neuronal marker βIII-tubulin and DAPI as described above. The images were taken with an LSM880 inverted confocal microscope (Carl Zeiss). Neuronal morphology, including total neurite length, branch number, and Sholl analysis, was quantified using the Simple Neurite Tracer (SNT) plugin from Fiji ImageJ, following the previous report (Ferreira et al., 2014). The iPSC-derived neurons (30 cells/each) were reconstructed after preprocessing the images into grayscale 8 bit format for enhanced tracing accuracy. Sholl analysis was executed from the soma, employing a maximum radial distance of 500 μm with 10 μm intervals between concentric Sholl circles.

### Microelectrode array electrophysiology

The NPCs (1.0 × 10^5^ cells/well) were transferred onto PLO/laminin-coated plates and cultured in BrainPhys Neuronal Medium for 1 week. The neurons were then cultured in normal neuron media or conditioned media of iMSCs for an additional 4 weeks. Spontaneous spikes were recorded for 10 min with MED64 Presto (Alpha MED Scientific) weekly starting 1 week after the differentiation. The spikes were detected when the recorded signal exceeded a threshold of ±5 *σ*, where *σ* was the standard deviation of the baseline noise during quiescent periods. The SBFs were detected using the four steps as previously described (Matsuda et al., 2018). First, spikes separated by interspike intervals of 20 ms were attributed to the same SBF. Datasets with a maximum number of spikes in the SBF below 20 spikes/SBF were eliminated from the analysis in the second step. SBFs separated by inter-SBF intervals shorter than 500 ms were combined in the third step. Finally, an SBF was defined when it contained over 500 spikes/SBF. The recorded spikes and SBFs were analyzed with MEA Symphony and Burst Analysis Table software (Alpha MED Scientific).

### Oxygen consumption rate and extracellular acidification rate measurement

The NPCs (3.0 × 10^4^ cells/well) were plated on PLO/laminin-coated Seahorse XF96 Cell Culture Microplate (Agilent) for differentiation into neurons and cultured in BrainPhys Neuronal Medium for 1 week. The neurons were then cultured in normal neuron media or conditioned media of iMSCs for additional 5 weeks. Isolated mouse synaptosomes (15 µg/well) were plated on polyethyleneimine-coated (PEI, Sigma-Aldrich) microplate and centrifuged at 3,000 × *g* for 1 h at 4°C to be attached to the plate. Oxygen consumption rate (OCR) and extracellular acidification rate (ECAR) were measured using Seahorse XFe96 Extracellular Flux Analyzer (Agilent) in accordance with the manufacturer's instructions in Seahorse XF Cell Mito Stress Test Kit (Agilent) or Seahorse XF Glycolytic Stress Test Kit (Agilent). The XF assay media of Mito Stress Test was made with glucose (10 mM for neurons and 15 mM for synaptosomes), glutamine (2 mM for neurons), and pyruvate (1 mM for neurons and 10 mM for synaptosomes), and the media of Glycolytic Stress Test was made with glutamine (2 mM). Oligomycin (2.5 µM for neurons and 5 µM for synaptosomes), FCCP (1 μM), and rotenone/antimycin A (0.5 μM) were used in Mito Stress Test, and glucose (10 mM), oligomycin (10 µM), and 2-deoxy-glucose (2-DG, 50 mM) were used in Glycolytic Stress Test. After OCR or ECAR was measured, the neurons were fixed and stained with DAPI, and the fluorescence intensity was measured using SpectraMax M5 (Molecular Devices) to assess the cell density. OCR and ECAR in each well were normalized by the cell density.

### Whole brain irradiation using X-Rad SmART micro-IGRT system and iMSC administration

Wild-type male C57/BL6 mice received whole brain irradiation (IR) using an X-Rad SmART micro-IGRT system under the anesthesia through isoflurane inhalation at 10 weeks of age. A cone-beam CT scan was performed, and the irradiator platform was adjusted to ensure proper positioning for radiation treatment. Whole brain irradiation was performed using a 10 × 10 mm collimator to deliver a focused dose of 15 Gy to the surface of the skin. The beam was positioned inferior to the olfactory bulb to reduce the risk of irradiating the eyes. The mice were administered with intravenous injection of iMSCs (1 × 10^6^ cells in 200 µl of 0.9% saline) or vehicle control (saline) through the lateral tail vein 24 h after the whole brain IR.

### Isolation of synaptosomes and radiation

Mouse synaptosomes were isolated from the mouse brain using Syn-PER Synaptic Protein Extraction Reagent (Thermo Fisher Scientific) according to the manufacturer's instructions. Briefly, the whole brain samples were homogenized in the Syn-PER Reagent with Protease and Phosphatase inhibitor Cocktail (Thermo Fisher Scientific). The samples were centrifuged at 1,200 × *g* for 10 min and the remaining supernatants were transferred. The supernatants were then centrifuged at 15,000 × *g* for 20 min, and the pellets of synaptosomes were resuspended with the Syn-PER Reagent and Protease and Phosphatase inhibitor Cocktail. OCR of freshly isolated synaptosomes (15 µg/each) were measured using Seahorse XFe96 Extracellular Flux Analyzer (Agilent). In some experiments, the freshly isolated synaptosomes were irradiated at 4 Gy using an XRad160 biological irradiator (Precision X-Ray) in the presence of either control medium or iMSC CM and subjected to the OCR measurement.

### PET/CT imaging using Perkin Elmer G8 PET/CT

All animal procedures were Institutional Animal Care and Use Committee (IACUC) approved. Each animal was placed into a rodent restrainer where ∼80 µCi of [F-18] fluorodeoxyglucose (^18^F-FDG) was administered in ∼150 µl of sterile saline via injection into the tail (caudal) vein. Then, mice were individually placed into an induction chamber using 2–3% isoflurane until anesthetized. Ophthalmic ointment was applied to each eye prior to the animal being placed into the imaging shuttle, where heat and maintenance anesthesia (2.5% isoflurane) were supplied. After a 60 min uptake period, the animal was transferred to the G8 micro-PET/CT system (Sofie Biosciences) imaging at the desired time points (baseline and 1, 7, 21, and 35 d after IR). Each mouse PET/CT data were acquired via a 10 min static scan. These images were reconstructed and analyzed using the MIM Software. For each micro-PET/CT scan, regions of interest were drawn over the whole brain, cerebellum, and liver to calculate mean standard uptake values (SUVmean) and normalize to liver (SUVmean cerebrum/SUVmean liver).

### Statistical analysis

All statistical analyses were performed using EZR software and GraphPad Prism 9. Comparisons were performed using two-tailed Student's *t* test, one-way ANOVA with Turkey's correction, or Wilcoxon test. *p* values <0.05 were considered statistically significant.

## Results

### Neurotrophic effects of iMSCs on iPSC-derived neurons

**Figure 1.**
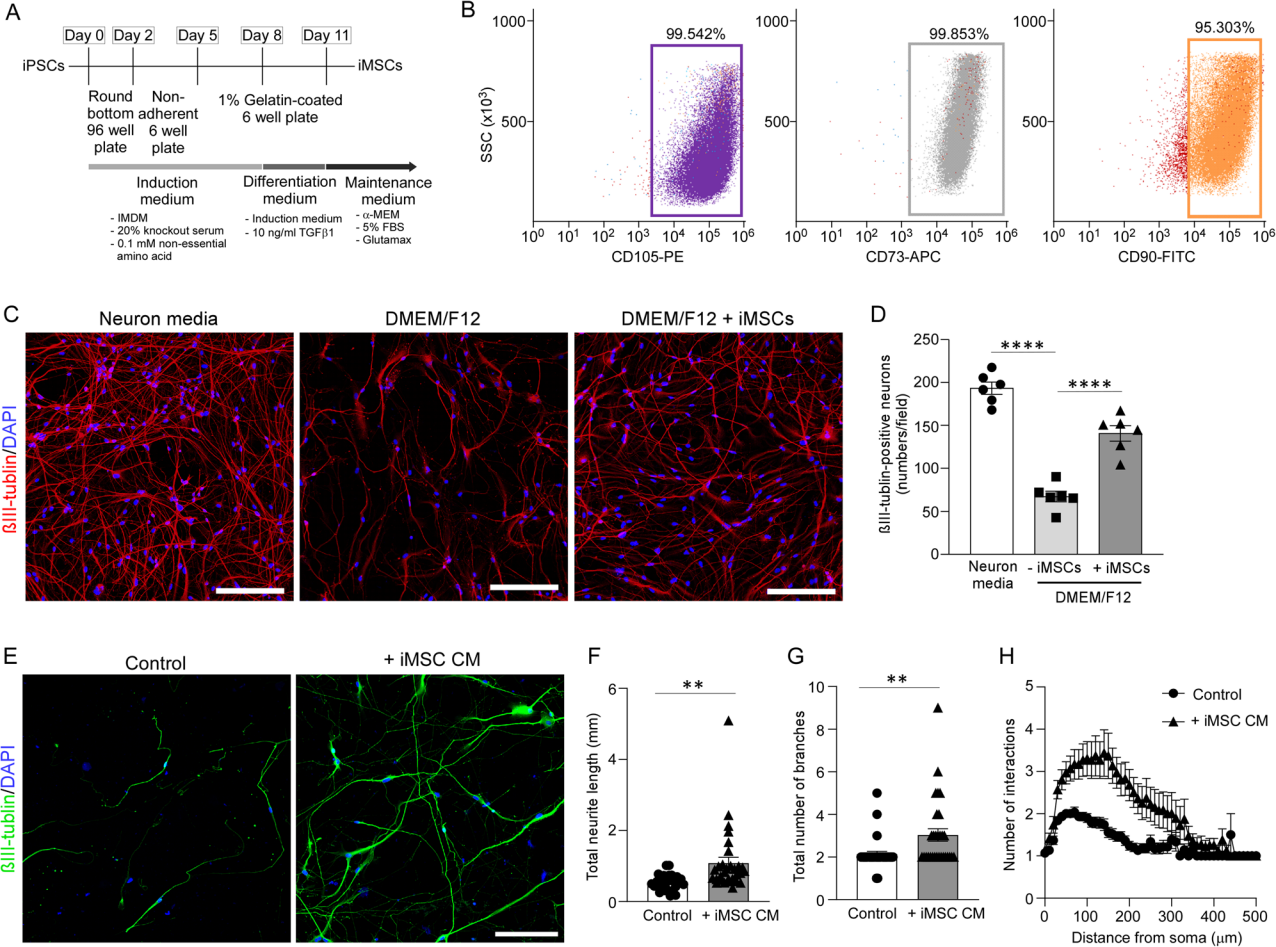
Conditioned medium from iMSCs induces neurotrophic effects on iPSC-derived neurons. ***A***, Schematic representation of the differentiation timeline from iPSCs to iPSC-derived MSCs (iMSCs). ***B***, Flow cytometry analysis showing that over 95% of iMSCs are positive for MSC markers CD105, CD73, and CD90. ***C***, The iPSC-derived neurons were cultured in the presence of normal neuron media, serum-free DMEM/F12 media, or cocultured with iMSCs for 1 week in serum-free DMEM/F12 media using a trans-well chamber from 1 week after the differentiation from NPCs. The iPSC-derived neurons were immunostained for βIII-tubulin. Nuclei were stained with DAPI. Scale bar, 200 µm. ***D***, The number of βIII-tublin-positive iPSC-derived neurons were quantified and normalized to cell number/field. Data represents mean ± SEM (***D***; *n* = 6 technical replicates/group). ***E***, The iPSC-derived neurons were cultured in normal neuron media or iMSC conditioned media for 3 weeks after differentiation from NPCs. The iMSC conditioned media was used from 1 week after the differentiation. iPSC-derived neurons were immunostained for βIII-tubulin. Nuclei were stained with DAPI. Scale bar, 200 µm. Total axon length (***F***) and branch number (***G***) of iPSC-derived neurons were quantified. ***H***, Sholl analysis was performed from the soma to 500 µm distance using a step size of 10 in the iPSC-derived neurons. Data represents mean ± SEM (***F–H***; *n* = 30 neurons/group) **p < 0.01, ****p < 0.0001 by one-way ANOVA with Tukey's correction (***D***), two-tailed Student's *t* test (***F***, ***G***), or ****p <0.0001 by two-tailed Wilcoxon test (***H***).

We differentiated iPSCs into iMSCs through a series of steps (Fig. 1*A*). The iMSCs were validated by flow cytometry, showing >95% positivity for MSC markers CD73, CD90, and CD105 (Fig. 1*B*). To investigate how iMSCs influence survival of iPSC-derived neurons, iPSC-derived NPCs were differentiated into mature neurons for 1 week, and then cocultured with or without iMSCs using trans-well chambers in serum-free DMEM/F12 medium for an additional week. Staining iPSC-derived neurons for the mature neuronal marker βIII-tubulin revealed a decrease in immunopositivity when cultured in serum-free DMEM/F12 medium compared with normal neuronal medium (Fig. 1*C*,*D*). However, the presence of iMSCs significantly elevated βIII-tubulin positivity in these neurons (Fig. 1*C*,*D*), suggesting that the iMSC secretome effectively promotes neuronal survival and growth. To further investigate the effects of iMSC secretome on neuronal growth, we utilized image reconstructions to analyze the structural features of iPSC-derived neurons following treatment with iMSC CM (Fig. 1*E*). After differentiating neural progenitor cells (NPCs) into neurons in maturation medium for 1 week, the neurons were subsequently cultured in either control medium or iMSC CM for an additional 3 weeks. Neurons treated with iMSC CM exhibited a significant increase in total neurite length and branch number compared with those treated with control medium (Fig. 1*F*,*G*). Additionally, we assessed neuronal dendritic complexity using Sholl analysis, which revealed a marked increase in intersections in neurons exposed to iMSC CM, indicating enhanced dendritic branching and complexity compared with control-treated neurons (Fig. 1*H*). These results collectively demonstrate that factors secreted by iMSCs promote neuronal viability, outgrowth, and dendritic architecture, suggesting that iMSC-derived neurotrophic molecules play a crucial role in supporting neuronal development and complexity.

### iMSC secretome enhances spontaneous synaptic activity in iPSC-derived neurons

**Figure 2.**
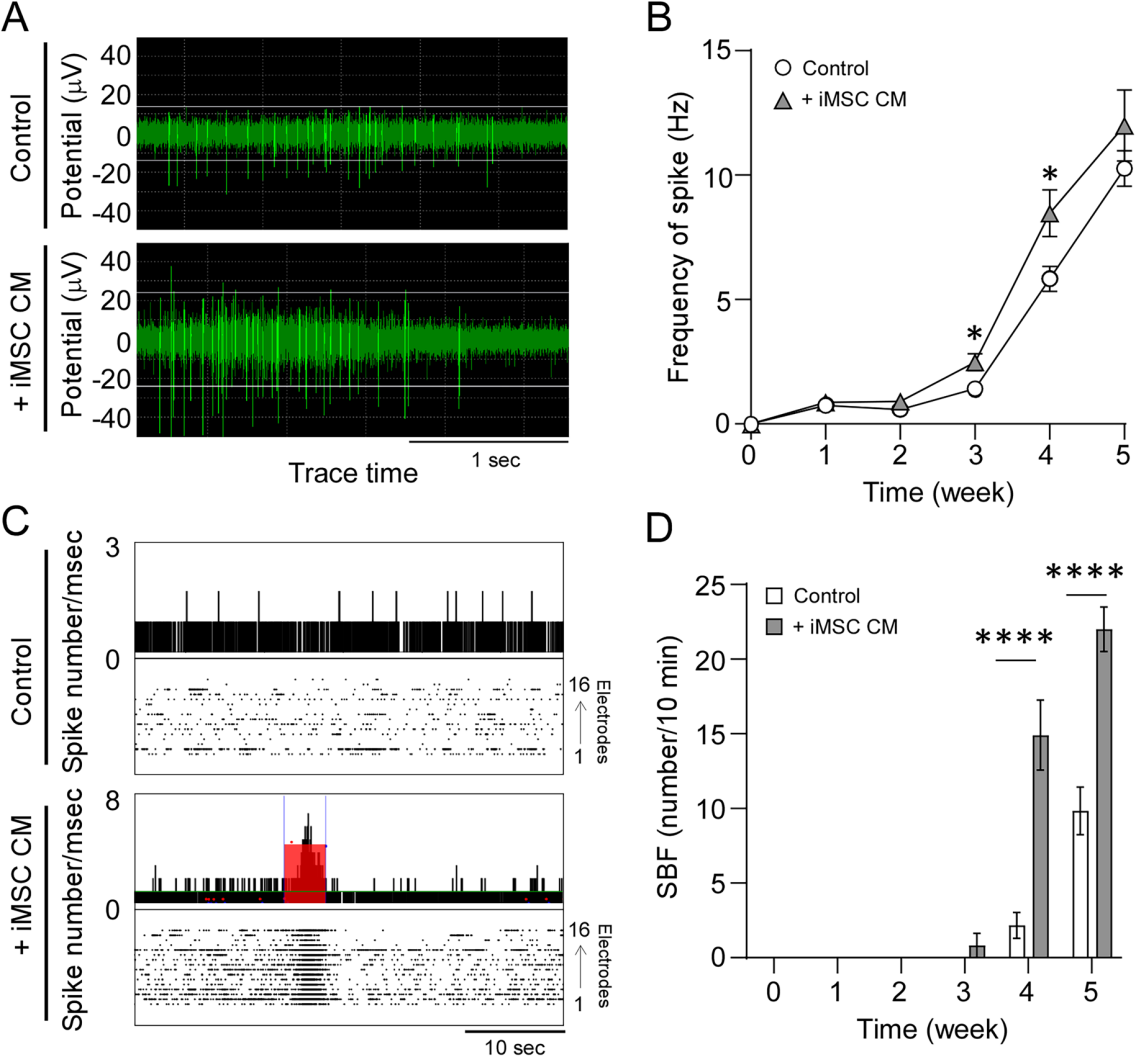
Conditioned medium from iMSCs accelerates spontaneous electrical activity and synaptic network formation in iPSC-derived neurons. ***A***, Spontaneous firing patterns in iPSC-derived neurons were measured 4 weeks after differentiation from NPCs in the presence of normal neuron media or iMSC conditioned media (iMSC CM). ***B***, The frequency of spontaneous firing was monitored in the iPSC neurons for 5 weeks after differentiation (*n* = 11–12 technical replicates/group). ***C***, Extracellular recordings of spontaneous firing including synchronized burst firing (SBF; red rectangles) in the iPSC neurons were measured 4 weeks after differentiation in the presence of normal neuron media or iMSC conditioned media. ***D***, The incidence of SBF was monitored in the iPSC-derived neurons for 5 weeks after the differentiation. Data represents mean ± SEM (*n* = 11–12 technical replicates/group). *p < 0.05, ****p < 0.0001 by two-tailed Student's *t* test.

To better understand the temporal functional effects of iMSC secretome on neuronal network formation, we turned to microelectrode array (MEA) electrophysiology, which records spontaneous electrical activity from multiple neurons simultaneously (McCready et al., 2022). NPCs were differentiated into neurons in maturation medium for 1 week, after which spontaneous synaptic firing and network formation were monitored weekly for an additional 4 weeks in the presence or absence of iMSC CM. Neurons treated with iMSC CM demonstrated a significant increase in spike frequency at 3 and 4 weeks compared with the control group, indicating enhanced synaptic activity and excitability (Fig. 2*A*,*B*). When we analyzed synchronized burst firing (SBF)—a critical parameter for effective neural network signal transmission—neurons treated with iMSC CM showed a pronounced increase in SBF at Weeks 4 and 5 relative to control-treated neurons (Fig. 2*C*,*D*). Collectively, these findings indicate that the iMSC secretome enhances synaptic network connectivity and accelerates the maturation of neuronal circuits.

### iMSC secretome boosts mitochondrial respiration and glycolysis in iPSC-derived neurons

**Figure 3.**
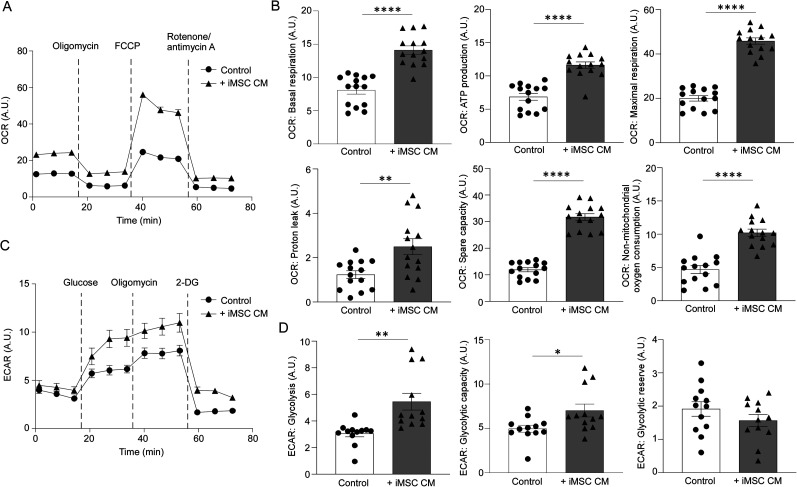
Conditioned medium from iMSCs activates mitochondrial respiration and glycolysis in iPSC-derived neurons. ***A***, ***B***, The oxygen consumption rate (OCR) in iPSC-derived neurons was measured in the presence of normal neuron media or iMSC conditioned media 6 weeks after differentiation from NPCs. The iMSC conditioned media (iMSC CM) was used from 1 week after the differentiation of iPSC-derived neurons. The OCR measurements were normalized with cell density determined by nuclear DNA staining with DAPI (*n* = 14 technical replicates/group). A.U., arbitrary unit. ***C***, ***D***, Glycolysis in the iPSC-derived neurons was measured in the presence of normal or iMSC conditioned media 6 weeks after the differentiation. The ECAR measurements were normalized with cell density determined by nuclear DNA staining with DAPI. Data represents mean ± SEM (*n* = 12 technical replicates/group). *p < 0.05, **p < 0.01, ****p < 0.0001 by two-tailed Student's *t* test.

Mitochondrial respiration and glycolysis are the major energy-yielding pathways in cells. Enhanced neuronal function—including efficient action potential generation, propagation, and synaptic transmission—is supported by improved neuronal metabolic activity (Sheng and Cai, 2012). Thus, we measured the effects of iMSC CM on the OCR, an indicator of mitochondrial respiration, in iPSC-derived neurons using Agilent Seahorse XF Analyzers (Bogetofte et al., 2019). NPCs were first differentiated into neurons over 1 week in neuron maturation medium and then cultured with control medium or iMSC CM for an additional 5 weeks before OCR measurements were conducted to evaluate mitochondrial activity. Administration of iMSC CM significantly increased basal respiration, maximal respiration, ATP production, and spare respiratory capacity of iPSC-derived neurons, indicating heightened baseline metabolic activity, enhanced mitochondrial capacity to produce energy under stress, and robust energy efficiency (Fig. 3*A*,*B*). Proton leakage in iPSC-derived neurons was also increased by iMSC CM, which may represent a regulatory mechanism to prevent the overproduction of reactive oxygen species (ROS) and protect the neurons from oxidative damage (Divakaruni and Brand, 2011; Fig. 3*B*). Additionally, non-mitochondrial oxygen consumption was significantly elevated in the presence of iMSC CM compared with the control group (Fig. 3*B*). These findings suggest that iMSC CM not only enhances mitochondrial respiration but also activates other metabolic pathways. We also found that basal glycolysis and glycolytic capacity, measured by the ECAR, were significantly increased in iPSC-derived neurons treated with iMSC CM, indicating an enhanced potential for energy production under high demand (Fig. 3*C*,*D*). However, iMSC CM did not affect the glycolytic reserve in iPSC-derived neurons (Fig. 3*D*). Together, these findings imply that the secretome from iMSCs enhances metabolic activity in neurons, improving neuronal growth and synaptic network function.

### iMSC secretome exerts neuroprotective effects in iPSC-derived neurons under stress conditions

**Figure 4.**
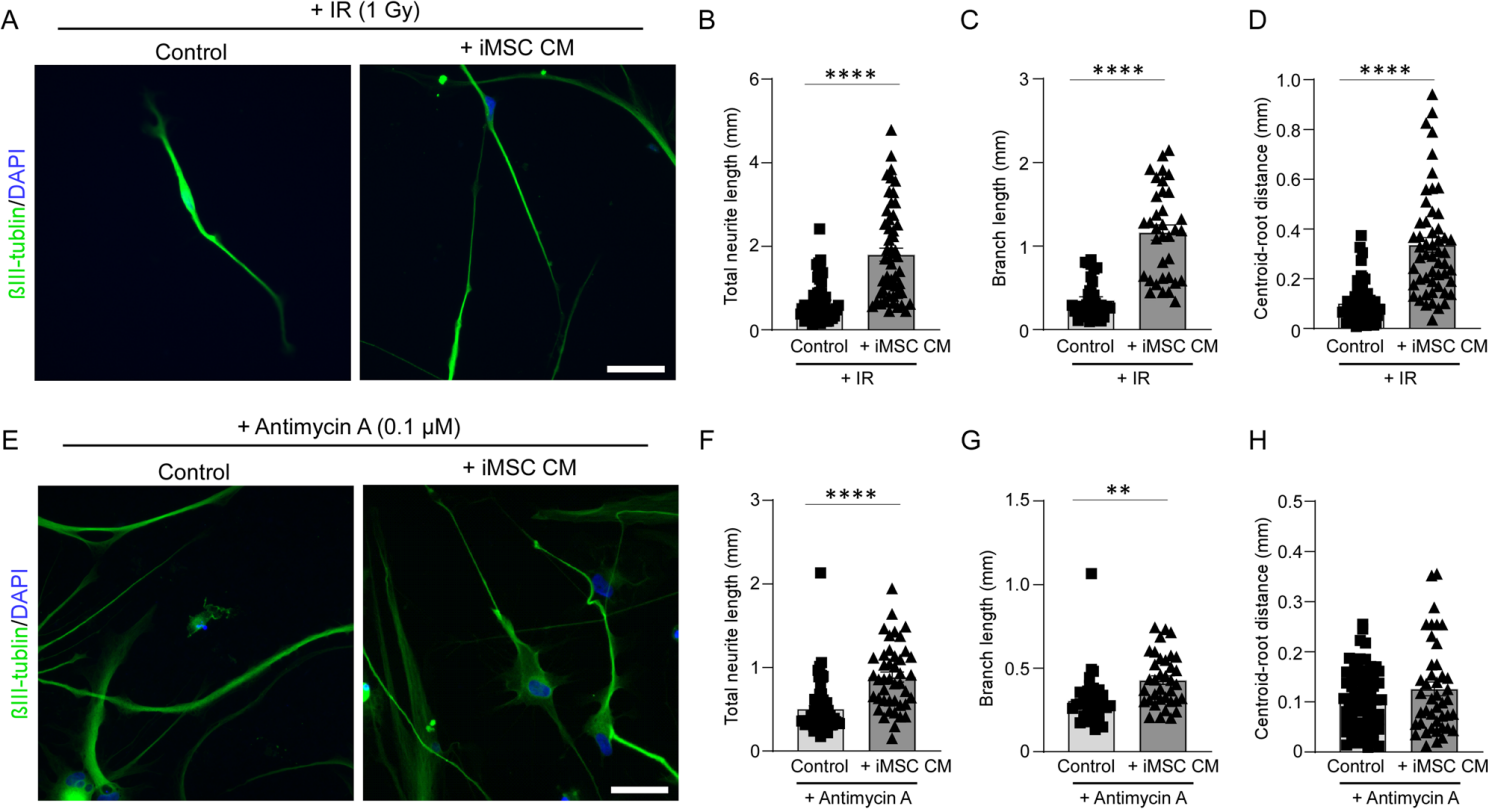
Conditioned medium from iMSCs improves neuronal outgrowth in damaged iPSC-derived neurons. ***A***, The iPSC-derived neurons were irradiated with a 1 Gy dose 1 week after the differentiation from NPCs in the presence of control media or iMSC conditioned medium (iMSC CM) for 5 d, followed by immunostaining for βIII-tubulin (green) and DAPI (blue). Scale bar, 50 µm. Total neurite length (***B***; *n* = 53–56 neurons/group), branch length (***C***; *n* = 35–36 neurons/group), and centroid–root distance (***D***; *n* = 53–56 neurons/group) were quantified in the irradiated iPSC-derived neurons treated with control media or iMSC CM. ***E***, The iPSC-derived neurons were cultured with a 0.1 µM antimycin A 1 week after the differentiation from NPCs for 2 weeks in the presence of control media or iMSC conditioned medium (iMSC CM), followed by immunostaining for βIII-tubulin (green) and DAPI (blue). Scale bar, 50 µm. Total neurite length (***F***; *n* = 43–64 neurons/group), branch length (***G***; *n* = 36 neurons/group), and centroid–root distance (***H***; *n* = 43–64 neurons/group) were quantified in the iPSC-derived neurons treated with control media or iMSC CM. Data represent mean ± SEM. **p < 0.01, ****p < 0.0001 by two-tailed Student's *t* test.

Radiation impairs neuronal functions by inducing acute disruptions in synaptic structures, which contribute to long-term cognitive deficits (Wu et al., 2012). To investigate the effect of iMSC secretome on neurons damaged by radiation, the iPSC-derived neurons were irradiated (1 Gy) 1 week after the differentiation from NPCs in the presence of either control medium or iMSC CM. Five days postirradiation, immunocytochemistry was performed to assess neuronal morphology. The results revealed that irradiated neurons treated with iMSC CM exhibited a significant increase in total neurite length, branch length, and centroid–root distance compared with neurons treated with control medium (Fig. 4*A–D*). This suggests that the iMSC secretome may offer protective and regenerative effects, promoting neuronal growth and structural complexity even in the face of radiation-induced damage. To investigate whether the iMSC secretome promotes neuronal outgrowth by enhancing mitochondrial activity, we treated iPSC-derived neurons with antimycin A (0.1 µM), a mitochondrial electron transport chain complex III inhibitor. This treatment was applied in the presence of either control medium or iMSC conditioned medium (CM), starting 1 week after differentiation from NPCs and continuing for 2 weeks. We found that iPSC-derived neurons treated with iMSC CM exhibited a significant increase in total neurite length and branch length compared with those treated with control medium even in the presence of antimycin A, suggesting that the iMSC secretome may promote neuronal outgrowth independently of mitochondrial electron transport chain complex III activation (Fig. 4*E–G*). No significant changes were observed in the centroid–root distance (Fig. 4*H*), implying that the iMSC secretome may influence the overall spatial orientation of the neuronal structures by improving mitochondria function.

### iMSC secretome increases mitochondrial respiration in synaptosomes from radiation-injured mouse brains

**Figure 5.**
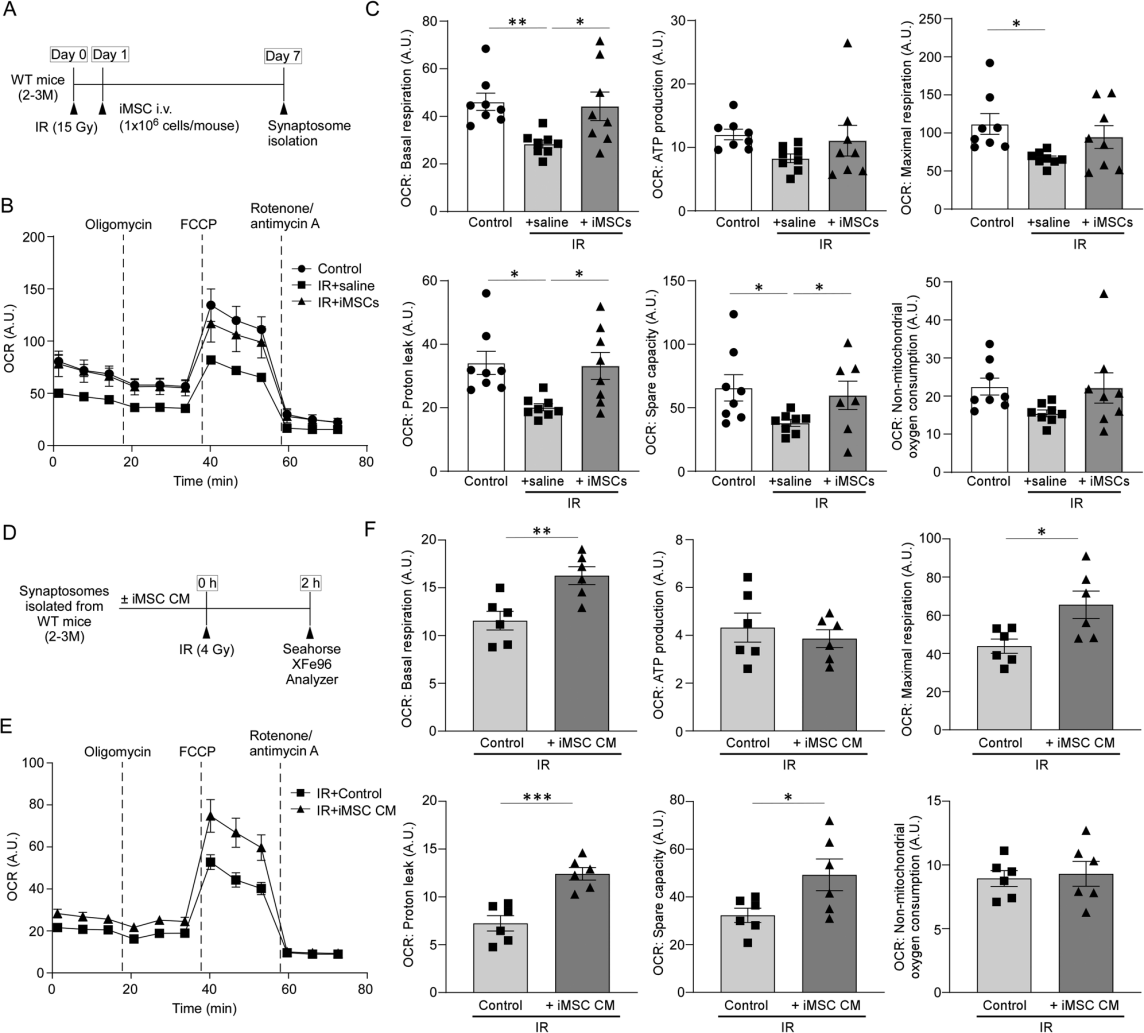
iMSC secretome ameliorates IR-induced impairment of mitochondrial respiration in mouse synaptosomes. ***A***, Male wild-type mice were treated with intravenous injection of saline (200 μl/each) or iMSCs (1 × 10^6^ cells in 200 μl saline/each) 24 h after whole brain irradiation (IR) at 15 Gy. The synaptosomes were isolated from the mouse brains 7 d after IR and subjected to mitochondria respiration analysis. ***B***, ***C***, Oxygen consumption rate (OCR) of synaptosomes (15 μg/each) was measured by Mito Stress Test Kit through Seahorse XFe96 Extracellular Flux Analyzer. Synaptosomes extracted from mouse brains without IR administration were used as controls. Data represents mean ± SEM (*n* = 8 technical replicates from 4 mice/group). ***D***, The synaptosomes were isolated from male wild-type mice, irradiated at 4 Gy in the presence of control media or iMSC conditioned medium (iMSC CM), and subjected to mitochondria respiration analysis. ***E***, ***F***, OCR of synaptosomes (15 μg/each) was measured 2 h after the IR. Data represents mean ± SEM (*n* = 6 technical replicates from 3 mice/group). A.U., arbitrary unit. *p < 0.05, **p < 0.01, ***p < 0.0001, ****p < 0.0001 by one-way ANOVA with Tukey's correction (***C***) or two-tailed Student's *t* test (***F***).

To explore the neuroprotective and regenerative effects of iMSCs on synaptic function, we utilized an in vivo mouse model subjected to whole brain radiation to model acute brain injury. This approach enabled us to closely examine the therapeutic potential of iMSCs in mitigating radiation-induced damage at the synaptic level. Wild-type male mice, at 10 weeks old, received whole brain irradiation (IR; 15 Gy), followed by an intravenous injection of iMSCs (1 × 10^6^ cells each) or vehicle control 24 h after the IR. Synaptosomes were isolated from mouse brains 7 d postinjury and subjected to OCR measurement (Fig. 5*A*). Radiation diminished basal and maximal respiration, ATP production, proton leak, spare respiratory capacity, and non-mitochondrial oxygen consumption in the synaptosomes. However, iMSC treatment effectively countered these radiation-induced reductions in OCR, specifically for basal respiration, proton leak, and spare respiratory capacity (Fig. 5*B*,*C*). To further explore whether iMSC secretome contributes to the enhanced synaptosome metabolic activity under mitochondrial stress, we conducted an additional experiment using isolated synaptosomes from wild-type mice. The freshly isolated synaptosomes were subjected to 4 Gy irradiation in the presence of control medium or iMSC CM (Fig. 5*D*). When mitochondrial respiration was investigated, we found that synaptosomes treated with iMSC CM exhibited a significant increase in OCR for basal respiration, maximal respiration, proton leak, and spare respiratory capacity compared with those incubated with control medium. There were no evident changes in ATP production or non-mitochondrial oxygen consumption (Fig. 5*E*,*F*). Consistent with the observations in whole brain irradiation mouse models treated with intravenous iMSC injection, these findings suggest that iMSC secretome is sufficient to mitigate neuronal injury by selectively enhancing synaptic energy metabolism.

### iMSC treatment boosts brain glucose metabolism in mouse models with radiation-induced brain injury

**Figure 6.**
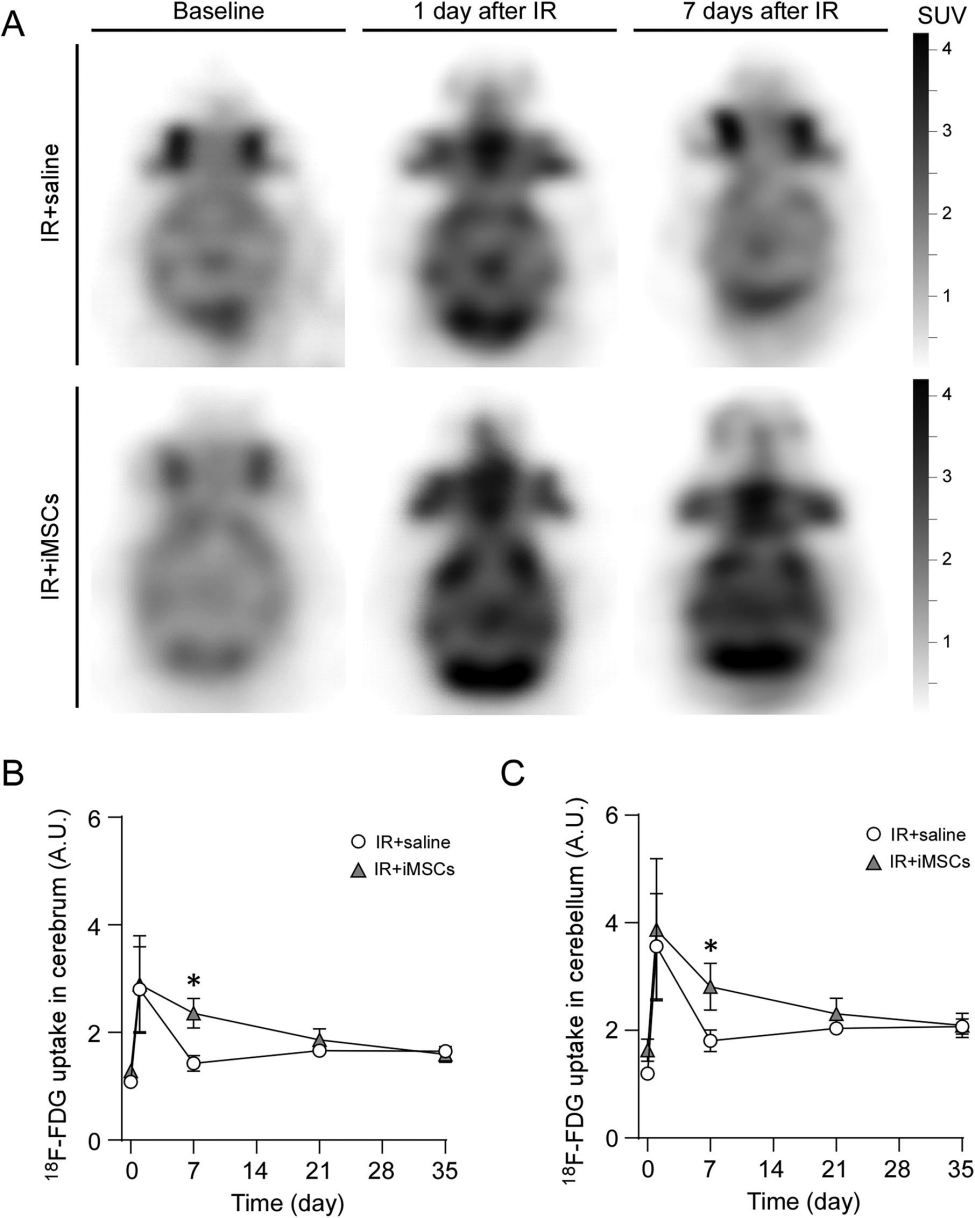
iMSC administration modulates brain glucose uptake after radiation-induced brain injury. Male wild-type mice were treated with intravenous injection of saline (200 μl/each) or iPSC-derived MSCs (iMSCs; 1 × 10^6^ cells in 200 μl saline/each) 24 h after whole brain irradiation (IR) at 15 Gy. ***A***, Representative ^18^F-FDG PET images of mouse brains before administration or IR (baseline) and at 1 and 7 d after irradiation were shown. These images demonstrate temporary increased ^18^F-FDG uptake at 24 h after irradiation (SUVmean of 3.1) with resolution to baseline at Day 7 (SUVmean of 2.2 and 2.5, respectively). Conversely, ^18^F-FDG PET in mouse brain treated with iMSC demonstrates persistently increased ^18^F-FDG at 24 and 7 d (SUVmean of 3.7 and 3.4, respectively) compared with baseline (SUVmean of 1.7). ***B***, ***C***, Summed ^18^F-FDG uptake in the cerebrum (***B***) and cerebellum (***C***) in the mice treated with saline or iMSC was measured at baseline and 1, 7, 21, and 35 d after the whole brain IR and normalized to that in liver at each time point. Data represents mean ± SEM (*N* = 5 mice/group). *p < 0.05 by two-tailed Student's *t* test.

We next investigated how iMSC treatment influences brain metabolic responses to acute radiation-induced injury in vivo. To this end, we assessed brain glucose metabolism by employing positron emission tomography (PET) scan imaging with the ^18^F-FDG radioactive glucose analog. Wild-type male mice received intravenous injections of iMSCs 24 h following whole brain IR at a dose of 15 Gy. The ^18^F-FDG tracer was administered intravenously to the mice, with uptake quantified before and at 1, 7, 21, and 35 d post whole brain IR (Fig. 6*A*). ^18^F-FDG uptake in both the cerebrum (Fig. 6*B*) and cerebellum (Fig. 6*C*) showed a temporary increase 1 d post-IR but returned to baseline levels by Day 7 post-IR in the control group. These findings suggest that iMSC administration influences brain glucose metabolism in response to brain injury, supporting the notion of an increased metabolic demand during the brain's repair and recovery phases.

## Discussion

In neurodegenerative disorders, mitochondrial dysfunction manifests as impaired ATP production and increased oxidative stress due to overproduction of ROS, all of which contribute to the degenerative process by damaging neuronal cells and synapses (Sheng and Cai, 2012; Schondorf et al., 2014; Wilson et al., 2023). We found that iMSCs increase the metabolic activity of iPSC-derived neurons via both mitochondria respiration and glycolytic pathways, implying that iMSC treatment boosts the overall energy production and metabolic flexibility of neurons, which is beneficial for their function and survival (Sheng and Cai, 2012). Brain injuries can trigger a series of adverse effects, including inflammation, oxidative stress, and altered blood flow, hindering the neurons’ capacity to fulfill the energetic requirements for repair (Wilson et al., 2023). Moreover, the metabolic flexibility of neurons—their ability to switch between glucose and alternative energy substrates—is crucial for maintaining neuronal function and survival under stress conditions. However, in neurodegenerative diseases, this metabolic flexibility is compromised, exacerbating neuronal vulnerability. For example, in Alzheimer's disease, brain glucose hypometabolism is a well-documented phenomenon, highlighting a shift in the energy metabolism of the brain that precedes cognitive decline (Costantini et al., 2008). We found that iMSC treatment ameliorates the detrimental impacts of acute brain injury on neuronal metabolism. Specifically, our observations imply an enhanced recovery in neuronal mitochondrial function and an increase in brain glycolytic activity in iMSC-treated mice after whole brain IR. This dual metabolic enhancement suggests that iMSCs not only support the immediate energy needs of damaged neurons but also contribute to establish a more resilient metabolic state, potentially facilitating better recovery and adaptation in the face of ongoing neurodegenerative challenges. Indeed, the features of neurons are intimately tied to their metabolic activity, as the energy demands of maintaining and modifying synaptic connections, propagating action potentials, and performing cellular repair are substantial (Sheng and Cai, 2012). Our results demonstrated that iMSC secretome not only increases neuronal firing rate but also enhances rhythmicity of the electrical activity in iPSC-derived neurons. Thus, iMSC treatment could enhance neuronal excitability and network coordination, potentially fostering better functional connectivity and communication among neurons, by improving mitochondria function.

Enhanced mitochondria metabolism has been known to improve neurite complexity, excitability, and synaptic function in human neurons (Iwata et al., 2023). Mitochondria dysfunction is also associated with the age-dependent reduction in neurite growth in mouse primary neurons (Sutherland et al., 2021). Interestingly, we found that the iMSC secretome could promote neuronal outgrowth even in the presence of a mitochondrial complex III inhibitor. Thus, the neuroprotective and neurotrophic effects of the iMSC secretome may be mediated by additional factors beyond its ability to increase mitochondrial metabolic activity. Neuronal health is overall supported by neurotrophic factors such as brain-derived neurotrophic factor (BDNF) and nerve growth factor (NGF), which promote survival, growth, and differentiation while facilitating synaptic plasticity (Hofer and Barde, 1988; Thoenen, 1995). Impaired neuronal form and function are hallmarks of neurodegenerative disorders such as Alzheimer's disease, Parkinson's disease, amyotrophic lateral sclerosis, and others (Ferraiuolo et al., 2011; Grienberger et al., 2012; Wei et al., 2013; Schondorf et al., 2014). MSCs, derived from different sources, display profound neurotrophic effects via multiple mechanisms including neurotrophic factor secretion, reduction of inflammation, delivery of exosomes packed with microRNAs and proteins, as well as the reduction of oxidative stress and an increase in synaptic plasticity (Zhang et al., 2017; Arakawa et al., 2023). Our previous study also found that iMSC secretome contains BDNF and hepatocyte growth factor (HGF) as well as proangiogenic and immunosuppressive factors (Gupta et al., 2024). Thus, our data serves as proof of concept that iMSCs provide a nurturing environment that not only secretes a range of neurotrophic factors but also supports the metabolic needs of neurons. Although further studies are needed to establish the identity of these factors, it is also possible that iMSCs transfer their mitochondria to damaged neurons to facilitate neuronal resilience and regeneration. Each of these provocative mechanisms are subject of future exploration.

In summary, our findings highlight the therapeutic potential of iMSCs in addressing the complex metabolic dysregulation associated with brain injury and neurodegenerative diseases, paving the way for novel regenerative strategies that target metabolic restoration and support key components of neuronal recovery and health maintenance. Future studies should aim to unravel the specific molecular pathways through which iMSCs exert their beneficial effects on neurons and their long-term impact, potentially unlocking new therapeutic avenues for a range of neurological disorders where current treatments are limited. Since iMSCs can be readily manufactured for long-term storage and scaled for use as an off-the-shelf regenerative biotherapeutic product, iMSC therapy could be a versatile tool in combating the multifaceted nature of degenerative conditions. Indeed, a phase I clinical trial showed the safety and efficacy of iPSC-derived MSCs (CYP-001) to treat acute steroid-resistant graft versus host disease (Bloor et al., 2020).
